# Environmental considerations in health technology assessments performed by Canadian agencies

**DOI:** 10.1017/S026646232510024X

**Published:** 2025-06-25

**Authors:** Elodie Bénard, Komi Edem Gatovo, Jason Robert Guertin

**Affiliations:** 1 Centre de recherche du CHU de Québec-Université Laval, Québec City, Québec, Canada; 2 Département de médecine sociale et préventive, Université Laval, Québec City, Québec, Canada

**Keywords:** health technology assessment, Canada, environment, review, guidelines

## Abstract

**Objectives:**

Globally, several health technology assessment (HTA) agencies have started to incorporate environmental considerations into their assessments, given healthcare systems’ substantial environmental footprint. In Canada, two HTA agencies, the Canadian Drug Agency and the Institut national d’excellence en santé et en services sociaux, have announced measures to help mitigate healthcare’s contribution to climate change. Our aim was to review reports from both agencies to identify those incorporating environmental considerations.

**Methods:**

We retrieved reports published between 1 May 2023 and 1 December 2024 by the two agencies.

**Results:**

We identifed 202 reports, of which eleven were included. These reports covered diverse technologies, with greenhouse gas emissions and waste production being the most frequently considered environmental dimensions. Parallel evaluation was the predominant method for integrating environmental considerations. We believe that the limited number of reports included may reflect the challenges of incorporating such considerations into HTAs.

**Conclusion:**

By addressing these challenges, HTA agencies could play a pivotal role in guiding decisions that align with environmental goals.

## Introduction

Health technology assessment (HTA) is a systematic evaluation process that examines the properties of a health technology, including both its direct and indirect impacts (1). HTAs can include a wide variety of data, regarding, for example, clinical, economic, social, ethical, legal, and environmental dimensions (2). They synthesize these data to provide decision-makers with a comprehensive overview that helps determine the optimal use of technologies in healthcare systems.

Healthcare systems, while dedicated to improving human health, have a significant environmental footprint (3;4). In 2019, they were estimated to be responsible for 4.4 percent of global greenhouse gas (GHG) emissions (3;4). If the healthcare sector were a country, it would be the fifth-largest emitter of GHG (4). In addition to their GHG emissions, healthcare systems’ energy-intensive facilities, water consumption, and waste production are increasingly drawing attention (4;5). As climate change continues to pose severe risks globally, evaluating the environmental impact of health technologies, such as carbon emissions, material resource utilization, and waste production, may enable policy decisions that, in addition to health outcomes and economic efficiency, also consider environmental sustainability.

Several HTA agencies across the world have started to incorporate environmental considerations into their assessments (6). In Canada, the Canadian Drug Agency (CDA) and, in Québec, the Institut national d’excellence en santé et en services sociaux (INESSS) have announced plans to implement new measures to help reduce healthcare’s contribution to climate change (6-8). Other agencies worldwide (e.g., in the United States, the United Kingdom, Australia, Sweden, Austria, and Colombia) have taken similar steps (6). Their approaches involve, for example, incorporating environmental objectives into their strategic planning and examining and integrating methodologies to quantify and report the environmental impact of health technologies.

Recently, a scoping review examined the approaches used to include environmental impacts in HTA (9). The authors identified three primary methods used. First, parallel evaluations that consider environmental impacts by calculating metrics such as an incremental carbon footprint effectiveness ratio or an incremental carbon footprint ratio. They may also consider environmental impact in the HTA decision-making process, including environmental impact in a multi-criteria decision analysis, or be conducted alongside an economic analysis. Second, evaluations that fully integrate environmental impacts into economic analyses, either as an outcome or a cost, or by adapting the willingness-to-pay threshold to reflect environmental impact. Third, environment-focused evaluations that concentrate solely on the environmental impact of a technology, without incorporating costs or health benefits.

By reviewing reports published by CDA and INESSS on the evaluation of health technologies that include environmental considerations, we analyzed how Canadian HTA agencies incorporate environmental impacts into their work and reports.

## Methods

### Data sources and selection process

We retrieved reports, identified as “Health Technology Review” (CDA) and “Avis” (INESSS), published by both agencies between 1 May 2023 and 1 December 2024. Identification of relevant reports to be included in our review was conducted by two reviewers, and any discrepancies were discussed until consensus was reached.

### Selection criteria

We included reports that incorporated environmental considerations, regardless of the technology assessed or the environmental dimension considered. We excluded reports that did not include any environmental aspect or were not fully published yet (e.g., reports identified as “in progress” by CDA).

### Data collection

We extracted data from the included reports using a standardized data collection form. Extracted data included information regarding the health technology assessed, the environmental dimension considered (e.g., GHG emission, energy consumption, and waste production), and how these considerations were included in the report. We classified the data and aggregated health technologies that belong to the same category.

### Methodological quality assessment

As our objective was to identify health technologies evaluated with environmental considerations and describe these considerations, we did not assess methodological quality.

## Results

**Figure 1.**
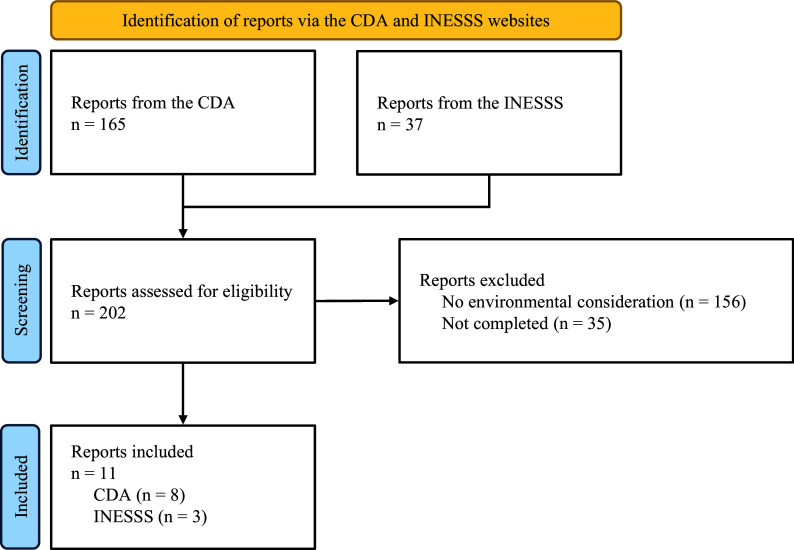
Flowchart. Flowchart for the report selection process.

We identified 202 reports (165 from CDA and thirty-seven from INESSS) published between 1 May 2023 and 1 December 2024 (Figure 1). We reviewed all reports and excluded 156 reports because they did not include any environmental consideration and thirty-five reports because they were not completed yet. We included eleven reports (eight from CDA and three from INESSS).

The included reports covered a wide range of technologies that can be classified into four main categories: clinical procedures; medical supplies and equipment; medications; and diagnostic tests (Table 1). Five reports addressed clinical procedures: short-cycle autoclave sterilization of instruments in same-day ophthalmic surgeries; minimum retesting intervals for lab tests; optimization of iodinated contrast media use; timing of ventilator circuit tubing replacement; and virtual care (10-14). The remaining reports were equally distributed among the other categories: two reports addressed medical supplies and equipment (nonsterile glove use and reprocessed single-use semi-critical and critical medical devices (15;16)); two reports addressed medications (inhaled anesthetic agents during general anesthesia and aerosol therapy with inhalers during mechanical ventilation (17;18)); and two reports addressed diagnostic tests (human papillomavirus virus testing (self-collected sample) and determination of blood titanium levels (19;20)).

**Table 1.** Characteristics of included reports

Category	Health technology	Agency	Environmental dimension considered	Inclusion of environmental considerations in the report
**Clinical procedures**				
	Short-cycle autoclave sterilization of instruments in same-day ophthalmic surgeries (10)	CDA	Carbon emissions and waste production	Discussion on the significant economic and environmental burdens potentially caused by using a fully wrapped, terminal steam sterilization cycle of ophthalmic instruments in autoclaves, and whether short-cycle sterilization of instruments could help reduce the environmental impact.
	Minimum retesting intervals for lab tests (11)	CDA	Carbon emissions and waste production	Discussion on the value of reducing unnecessary lab testing, emphasizing that unnecessary repeat testing contributes to environmental harm through carbon emissions and environmental waste.
	Optimizing the use of iodinated contrast media (12)	CDA	Water contamination	Discussion on reevaluating standard practices regarding the use of iodinated contrast media (ICM), considering the environmental concerns about water contamination associated with ICM and the potential costs savings from reducing its usage.
	Timing of ventilator circuit tubing replacement (13)	CDA	NA	Discussion on advocating for the adoption of a sustainable choice of devices that are both clinically effective and cost-effective, while also minimizing environmental impact.
	Virtual care (14)	CDA	NA	Environmental factors (e.g., carbon emission changes due to reduced travel) were among the implementations considerations examined. However, the authors found no scoping reviews that discussed environmental or climate considerations for virtual care use.
**Medical supplies and equipment**				
	Nonsterile glove use (15)	CDA	Waste production	Discussion on the implications of excess glove use, including its role in increasing medical waste and contributing to environmental contamination.
	Reprocessed single-use semi-critical and critical medical devices (16)	CDA	NA	Discussion on the growing interest in evaluating the clinical safety of reprocessed single-use medical devices, given the potential economic and environmental benefits of their reuse.
**Medications**				
	Inhaled anesthetic agents during general anesthesia (17)	INESSS	GHG emissions	Social costs associated with carbon footprint included in economic evaluation. Economic analysis based on data from Quebec showed that the use of desflurane is inefficient compared to isoflurane and sevoflurane, as it leads to increased acquisition costs and higher social costs associated with carbon footprint.
	Aerosol therapy with inhalers during mechanical ventilation (18)	CDA	GHG emissions	Discussion on the importance of recognizing the environmental footprint of metered dose inhalers (MDIs) due to their greenhouse gas emissions. Given their notable carbon emissions, it is crucial to ensure that MDIs are used appropriately and only when necessary.
**Diagnostic tests**				
	Human papillomavirus virus testing (self-collected sample) (19)	INESSS	Waste production	Discussion on the distribution methods of kits (via postal service, pharmacy, and health center) to limit the number of kits distributed but not used.
	Determination of blood titanium levels (20)	INESSS	NA	Discussion on the fact that the determination of blood titanium levels is currently performed outside of Quebec. Local availability of the test could reduce sample transportation and thus offer environmental benefits.

The most common environmental dimensions considered were GHG emissions and waste production. Six reports considered GHG emissions (including carbon emissions) and/or waste production (GHG emissions in two reports (17;18), waste production in two reports (15;19), and both carbon emissions and waste production in two reports (10;11)). One report mentioned water contamination (12), and the four remaining reports discussed environmental impact but did not identify a specific environmental dimension (13;14;16;20).

In nine reports, the approach used to include environmental considerations associated with the technology was a parallel evaluation (10-13;15;16;18-20). These reports considered environmental impacts during the HTA deliberation process and/or reported and discussed these impacts alongside other data and analyses (e.g., economic evaluation). Only one report, addressing inhaled anesthetic agents during general anesthesia, included an integrated evaluation (17). In that report, environmental data were incorporated into an economic evaluation using the social cost of carbon associated with the different anesthetic agents examined. In the report addressing virtual care, environmental impacts were among the implementation considerations examined (14). However, the authors found no data regarding the environmental or climate considerations for virtual care use and, therefore, were unable to report environmental impacts.

## Discussion

Among the 202 identified reports, only eleven included environmental considerations, showing that environmental impacts are rarely incorporated into HTAs. However, it is important to keep in mind that if environmental aspects were considered during the report’s preparation but not included in the final report (e.g., due to feasibility reasons), we were unable to capture this information and include those reports, as we only included final published reports that integrated environmental considerations. In the included reports, we noticed a predominant focus on GHG emissions and waste production, and that parallel evaluation was the most used approach for incorporating environmental impacts.

Our findings align with those of several studies (9;21;22). Although these studies did not focus specifically on HTAs performed by Canadian agencies, they found that the integration of environmental considerations in HTAs is still in its early stages (22), that GHG emissions are among the most commonly assessed environmental impacts in HTAs (9;21), and that HTAs often assess environmental impacts separately rather than integrating them fully into economic analyses (9).

We believe that the small number of reports including environmental considerations reflects the difficulty of incorporating these considerations into HTAs. When it comes to evaluating the environmental impacts of a technology, agencies face numerous challenges. For example, while environmental considerations would, ideally, be included for all assessed technologies, the relevance of incorporating these considerations in an HTA may vary depending on the technology (21;23). Determining which health technologies would benefit the most from an evaluation of their environmental impact and establishing the appropriate scope of such an assessment could be complicated (9;21). Moreover, the methods for including environmental impacts are complex, especially in the case of integrated evaluations, and there is limited guidance on which one should be used in a specific context (9;24). There is a pressing need to determine the most appropriate methods to use, depending on the technology being evaluated and the relevance to integrate environmental considerations for this specific technology. Over time, these methods could be refined and standardized to ensure that environmental considerations are consistently integrated in HTAs when appropriate (21). Finally, other challenges, such as the lack of relevant environmental data and the limited resources (both in terms of personnel and funding) relative to the volume of technologies that must be assessed, may also complicate the integration of environmental considerations into HTAs (6;21;24).

Both Canadian agencies are actively working to enhance the integration of environmental considerations into HTAs. For example, CDA’s 2022–2025 strategic plan outlines methodological adaptations to integrate environmental sustainability (7). The agency adopted a life-cycle approach to HTA, enabling assessments of a technology’s environmental impact at various stages, from research and development to after it has been replaced by a newer technology (7). Regarding INESSS, the agency recently published a bulletin on the environmental impacts in health and social services, which provides an overview of the methodological options for incorporating environmental considerations throughout the HTA process (6). INESSS also plans to report its progress in sustainable development to its board of directors or through institutional action plans, and to raise awareness or train personnel on climate issues (6;25). In addition, both agencies have expressed their intention to incorporate sustainability into prioritization criteria, examine existing methods and frameworks used to integrate environmental impacts to improve assessment methods, and develop deliberative frameworks that address environmental dimensions (6;8).

## Conclusion

Few reports from CDA and INESSS included environmental considerations. Although several initiatives are underway, methods for evaluating and integrating environmental considerations into HTAs remain complex, and there is little guidance on which approach to adopt. By addressing these issues, both agencies could play a pivotal role in guiding decisions that align with broader social and environmental goals and could, therefore, help reduce the environmental impact of health technologies used in healthcare.
